# Dual-functionalized lignin as a sustainable modifier for high-performance phenol-formaldehyde adhesives in plywood production

**DOI:** 10.1039/d5ra04233j

**Published:** 2025-09-05

**Authors:** Nadia Anter, Ahlam Chennani, Mohamed-Yassine Guida, Fatima ezzahra Atmani, Amine Moubarik, El Mostapha Rakib, Abdelouahid Medaghri-Alaoui, Abdellah Hannioui

**Affiliations:** a Molecular Chemistry, Materials and Catalysis Laboratory, Faculty of Sciences and Techniques (FST-BM), University of Sultan Moulay Slimane (USMS) Béni-Mellal 23000 Morocco nadia.anter@usms.ma; b Environmental, Ecological and Agro-Industrial Engineering Laboratory, Faculty of Sciences and Techniques (FST-BM), University of Sultan Moulay Slimane (USMS) Béni-Mellal 23000 Morocco; c Laboratory of Chemical Processes and Applied Materials, Polydisciplinary Faculty, Sultan Moulay Slimane University BP 592 Beni-Mellal Morocco; d Higher School of Technology, Sultan Moulay Slimane University BP 336 Fkih Ben Salah Morocco; e Department of Chemistry and Environment, Faculty of Sciences and Techniques (FST-BM), University of Sultan Moulay Slimane (USMS) Béni-Mellal 23000 Morocco

## Abstract

Biopolymers derived from natural sources are sustainable, non-toxic, and biodegradable, making them attractive alternatives to fossil-based polymers. Among these, lignin has garnered significant attention due to its potential in adhesive applications. In this study, lignin was extracted from redwood (*Pinus sylvestris* L.) sawdust using an alkaline delignification process and subsequently modified through propargylation and silanization. Comprehensive characterization using FTIR, solubility tests, TGA/DTG, ^1^H NMR, SEM-EDX, and contact angle measurements confirmed significant improvements in physicochemical properties. Notably, silylated lignins exhibited enhanced solubility in low-polarity solvents and a marked increase in hydrophobicity, with water contact angles reaching 127.7°. Phenol-formaldehyde (PF) adhesives incorporating raw and modified lignins (5–15 wt%) were formulated and applied in plywood production. Mechanical testing revealed that plywood bonded with 10 wt% Prop-lignin-DH achieved a bond strength of 5.44 MPa and 100% wood failure, outperforming conventional lignin-based adhesives. Furthermore, compared to the control PF resin, the modulus of rupture (MOR) and modulus of elasticity (MOE) increased by 38% and 27.3%, respectively. Shear strength also improved significantly, with gains of 56.1% under dry conditions, 17.6% in cold water, and 74.1% after boiling. In addition, formaldehyde emissions were reduced by 20% compared to standard PF resins, highlighting the potential of silylated lignin as a sustainable adhesive modifier for wood-based composites.

## Abstract 2

Dual-functionalization of lignin by propargylation and hydrosilylation enables its integration into phenol-formaldehyde adhesives, improving the performance of plywood panels.

## Graphical abstract

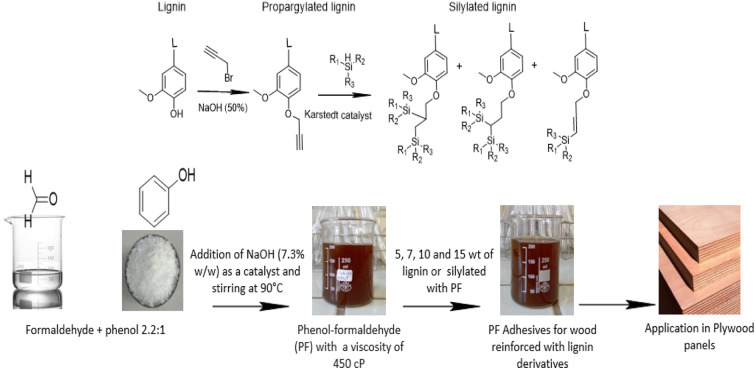

## 1. Introduction

Lignin is a complex polyphenolic macromolecule abundantly present in the cell walls of plants and constitutes one of the main structural components of wood, along with cellulose and hemicelluloses. It is an aromatic biopolymer biosynthesized through the radical polymerization of three primary monolignols: *p*-coumaryl alcohol, coniferyl alcohol, and sinapyl alcohol. These monomers give rise to a highly branched and irregular three-dimensional network interconnected through various ether and carbon–carbon linkages.^1^

Over the past decades, numerous lignin extraction techniques have been developed, including the kraft process, steam explosion, enzymatic hydrolysis, organosolv processes, alkaline delignification, and lignosulfonate recovery.^2^ Among these, alkaline treatment is widely recognized for its cost-effectiveness, simplicity, and reduced environmental impact.^3^

Despite its availability and chemical functionality, the industrial utilization of lignin remains limited due to several factors, including its complex and heterogeneous structure, dark coloration, low solubility in most solvents, broad molecular weight distribution, and variability in physicochemical properties.^2,4^ Its solubility in organic solvents is largely influenced by parameters such as molecular weight, chemical composition, and aliphatic hydroxyl group content.

Although lignin exhibits good compatibility with polar polymer matrices, its incorporation into nonpolar systems is often hindered by interfacial incompatibility, which negatively impacts the mechanical performance of the resulting materials. Nevertheless, the presence of reactive hydroxyl groups along the lignin backbone offers multiple opportunities for chemical modification aimed at improving its hydrophobicity, compatibility with apolar polymers, and dispersion within polymer matrices.^5,6^ To address these challenges, several functionalization strategies have been investigated, including methylation, alkylation, acetylation, propargylation, and silylation. These approaches have shown promise in enhancing lignin's solubility, chemical reactivity, and overall suitability for advanced material applications.^7^

Among these strategies, propargylation involves the introduction of propargyl groups onto the phenolic hydroxyl moieties of lignin *via* an SN_2_ reaction mechanism, significantly enhancing its chemical reactivity and broadening its application potential.^8–11^ This functionalization step serves as a key intermediate, offering access to a wide range of derivatives due to the high reactivity of the terminal alkyne group (–C≡C–). For instance, propargylated lignin can undergo hydrosilylation reactions with hydrosilanes (H–SiR_3_), forming robust Si–C bonds and enabling the development of functionalized lignin-based materials and advanced composites.

Hydrosilanes such as triethylsilane (Et_3_SiH) and polymethylhydrosiloxane (PMHS) have mainly been employed in the reductive cleavage of lignin model compounds, particularly targeting α-O-4 and β-O-4 linkages to generate phenolic and alcoholic products under mild conditions.^12,13^ However, their use has traditionally focused on depolymerization. In contrast, recent studies have highlighted the role of silane reagents—including *tert*-butyldimethylsilyl chloride,^14^ 1,1,3,3-tetramethyl-1,3-divinyldisilazane,^15^ aminopropyltrimethoxysilane,^16,17^ vinyltrimethoxysilane^18^ and methyltrimethoxysilane (MTMOS)^19,20^ —in enhancing lignin's solubility, thermal stability, mechanical performance, and compatibility with polymer matrices.

Propargylation targets the hydroxyl groups responsible for lignin's hydrophilicity, thereby reducing its affinity for water and increasing its hydrophobicity. Additionally, it introduces alkyne groups, enabling subsequent hydrosilylation by hydrosilanes. The latter further enhances hydrophobicity while improving lignin's thermal stability, polymer compatibility, and mechanical properties. Thus, the combination of these two modifications doubles the hydrophobic effect and expands the potential applications of lignin in advanced materials.

The increasing demand for polymer resins in civil and industrial applications has led to the widespread use of formaldehyde-based adhesives, such as melamine-urea-formaldehyde (MUF), urea-formaldehyde (UF), and phenol-formaldehyde (PF) resins, particularly in the wood panel industry. Among these, PF resins are especially favored due to their excellent mechanical strength, thermal stability, and flame resistance.^21,22^ However, they present a major drawback: the emission of formaldehyde, a known carcinogen.^23,24^ This issue has prompted intensive research into the development of renewable, formaldehyde-free adhesives that maintain performance comparable to their synthetic counterparts. Current strategies include fully bio-based adhesives composed of natural polymers and semi-synthetic systems that incorporate biopolymers into traditional resins.^25–29^ In this context, lignin has attracted considerable attention due to its aromatic structure, high availability, and favorable properties such as biodegradability, antioxidant activity, and antimicrobial effects. Its structural similarity to phenol makes it a suitable candidate for partially or fully replacing phenol in PF resins, or for cross-linking with formaldehyde.^30,31^

Despite this potential, the adoption of lignin in adhesive formulations is often hindered by its poor reactivity, structural heterogeneity, and limited water resistance, which compromise bonding performance. To address these challenges, Jedrzejczyk *et al.* developed lignin-based resins for wood bonding by using Protobind 1000, a commercial lignin product, combined with methanol-soluble fractions obtained by mild solvolysis. Through the introduction of terminal alkyne groups, the lignin was successfully cross-linked with multifunctional thiols, yielding adhesives with performance on par with PF resins and offering advantages such as higher lignin content and the absence of volatile emissions during processing.^8^ Similarly, Pau *et al.* demonstrated that magnesium lignosulfonate, when combined with diphenylmethane diisocyanate and glucose, significantly enhanced the mechanical properties of particleboard and reduced formaldehyde emissions.^32^ Peng *et al.* reported the formulation of a formaldehyde-free adhesive based on oxidized lignin and polyethylenimine (PEI), which met industrial performance standards for poplar panels.^33^ Younesi-Kordkheili *et al.* modified lignin with maleic anhydride, achieving improvements in both mechanical properties and formaldehyde reduction in LPF and UF resin systems.^34,35^ In a related study, lignin nanoparticles were modified using a deep eutectic solvent (ChCl–ZnCl_2_), allowing replacement of up to 70% of the phenolic component in lignin-phenol-formaldehyde resins. The resulting adhesives exhibited enhanced bonding strength and reduced formaldehyde content, while complying with international standards.^36^ Moreover, Iswanto *et al.* incorporated lignosulfonate into eco-friendly adhesive systems, improving the thermal stability and fire resistance of wood-based panels.^37^

To address the limitations of lignin in adhesive formulations—such as low reactivity, hydrophilicity, and structural variability—this study proposes a sequential dual-functionalization approach combining propargylation and hydrosilylation. In this strategy, propargyl groups are first introduced to the phenolic hydroxyls of lignin, replacing hydrophilic functionalities with terminal alkynes that serve as active sites for further reaction. Subsequent hydrosilylation with hydrosilanes such as dimethylphenylsilane (Me_2_PhSiH), 1,1,3,3-tetramethyldisiloxane (DH), introduces Si–C bonds that enhance thermal stability, hydrophobicity, and interfacial compatibility with polymer matrices. This method diverges from traditional reductive uses of hydrosilanes, which often lead to lignin depolymerization, by preserving the polymeric structure while improving its functional performance. The modified lignin was incorporated into phenol-formaldehyde (PF) adhesives at various loadings (0, 5, 7, 10, and 15 wt%), and the resulting formulations were evaluated through comprehensive physicochemical and mechanical testing. This dual modification significantly improves the adhesive performance and environmental profile of PF systems, highlighting lignin's potential as a sustainable alternative in wood-based composite applications.

## 2 Materials and methods

### 2.1 Materials

The redwood (RW) sawdust samples were collected in the Beni-Mellal area of the central zone (Morocco). The RW biomass samples utilized in this investigation came from the redwood species *Pinus sylvestris* L. Particle sizes ranging from 0.10–0.20 mm were obtained by crushing and sieving the materials. To evaluate the percentages, TGA analysis was employed. Fiber analysis by weight showed that 29.07% of the sample was hemicellulose, 42.51% was cellulose, 19.66% was lignin, and 8.76% was extractives.^38^

All chemical reagents used in the research were acquired from commercial sources. Propargyl bromide purity >97%, dimethylphenylsilane Me_2_PhSiH (>98.5%), 1,1,3,3-tetramethyldisiloxane DH (97%), karstedt's catalyst (Pt 2% in xylene), sodium hydroxide (NaOH, >97%), sulfuric acid (H_2_SO_4_) (97%), chloroform and benzene were sourced from Sigma Aldrich.

### 2.2 Methods of analysis

#### 2.2.1 Fourier transform infrared spectroscopy

A JASCO FT/IR 4600 Type A Fourier-Transform Infrared Spectrophotometer with an ATR accessory was used, the FTIR analysis of lignin samples was evaluated. A total of 16 scans were acquired for each sample during tests carried out in transmittance mode at ambient conditions and in the wavenumber range of 400 cm^−1^ to 4000 cm^−1^ with a resolution of 4 cm^−1^.

#### 2.2.2 Thermogravimetric analysis (TGA/DTG)

The thermal stability of the materials was assessed using a thermogravimetric analyzer (TGA) with the LABSYS evo TGA 1600 from SETARAM Instruments. Samples weighing between 2 and 20 mg were heated from 30 °C to 700 °C at a rate of 10°C min^−1^ under atmospheric pressure. The rate of weight change was evaluated through DTG-derived thermogravimetric analysis, which is the first derivative of TGA data. All thermal analysis measurements were performed under atmospheric conditions using the SETARAM LABSYS evo (TGA/DSC 1600).

#### 2.2.3 Scanning electron microscopy (SEM)

The morphology of samples was studied with the aid of a scanning electron microscope (SEM, Zeiss Evo 10) operating at 15 kV. Prior to SEM analysis, a thin conductive gold layer was applied to the samples using an ion sputtering device, after which an energy-dispersive X-ray (EDAX) analyzer was used to assess the elemental composition and distribution.

#### 2.2.4 Solid-state nuclear magnetic resonance spectroscopy

A Bruker Avance III spectrometer operating at 500 MHz, *z*-gradient probe head for inverse detection and DMSO-*d*_6_ solvent was used to record ^1^H NMR spectra. The alterations in chemical composition are expressed in *δ*-values [ppm] and are compared to the residual signals of DMSO-*d*_6_ (*δ* 3.3).

#### 2.2.5 Water contact angle measurements

The sessile drop technique was applied for the measurement of contact angles. After being examined, the lignin samples were pulverized into a fine powder and formed into a pellet. A HAMA AC-150 (0.3 MP) camera recorded the drop profile of distilled water that was applied to this particle. For every sample, at least 30 measurements were made.

#### 2.2.6 Solubility measurements

Each sample's solubility in both organic and aqueous solvents was determined using the procedure indicated by Sameni *et al.*^39^ In order to maintain the concentration at 3 g L^−1^ in each solution, the samples were separately added to organic solvents such as DMSO, ethanol, methanol, THF, and chloroform, as well as 1 M NaOH aqueous solution. The solubility tests were conducted with continuous stirring at 150–200 rpm for 20 minutes, maintaining a constant temperature of 25 °C. When the solubility tests were finished, the insoluble fractions were separated using Whatman no. 4 filter paper, then dried for two hours at 60 °C, weighed, and the samples' relative solubility was determined. Each experiment was carried out in triplicate, and the standard deviation (±) of the mean values was given. Ultimately, the solubility data were examined, and a plot of main effects was created to show how the sample solubility changed on average in response to different modification strategies.

#### 2.2.7 Statistical analysis

The means of the data and standard deviations were calculated, followed by an analysis of variance (ANOVA) conducted at a 5% significance level using OriginLab software (version 2019b 9.65, Massachusetts, USA). A Tukey test was applied for pairwise comparisons to identify significant differences.

### 2.3 Lignin extraction

Extraction of lignin was carried out using the methodology previously outlined by Mennani *et al.* et Akhramez *et al.*^19,21^ In brief, a two-liter beaker was filled with 50 g of sawdust. A solid/liquid ratio of 1 : 10 (w/v) was applied, and the sample was exposed to hot water at 70 °C for two hours. Following this process, the sample was cooled to 25 °C, followed by washing with water at a 1 : 10 (w/w) ratio to remove the solubilized hemicelluloses. After recovery, the solid residue was dried for 24 hours at 60 °C. A 15% w/v aqueous NaOH solution at a solid/liquid ratio of 1 : 10 (w/v) was used to treat the sample, followed by stirring for 4 hours at 98 °C. The black liquor was filtered, and the filtrate was acidified with 5 N sulfuric acid to reduce the pH to 2. After cooling to 25 °C, lignin precipitation was allowed to occur. The raw lignin was collected, washed with distilled water until the wash water reached a neutral pH of 7, and air-dried. Lignin yield was calculated using the formula:*Y*_L_ = *W*_1_/*W*_2_

The recovered lignin's mass (g) is denoted by *W*_1_, while *W*_2_ refers to the mass (g) of the sample subjected to extraction. A yield of 11 wt% was obtained.

### 2.4 Lignin modification

#### 2.4.1 Lignin propargylation

Lignin was modified *via* propargylation following the protocol reported by Dobrynin *et al.*,^40^ with slight modifications. Briefly, 2.00 g of kraft lignin was swollen in 5 mL of 50% aqueous NaOH solution (12.5 mol L^−1^) and stirred for 2 hours at 5 °C to ensure complete activation of phenolic hydroxyl groups. Subsequently, 7 mL (91.5 mmol) of propargyl bromide was added dropwise under constant stirring, followed by 50 mL of benzene as an organic solvent to facilitate the phase–transfer reaction. The mixture was maintained at 60 °C for 24 hours under atmospheric pressure. Upon completion, the reaction mixture was poured into a large excess of distilled water to precipitate the product. The precipitate was thoroughly washed with acetone and distilled water to remove unreacted reagents and by-products. The resulting propargylated lignin (Prop-lignin) was air-dried at 40 °C for 24 hours. No pH adjustment was necessary post-reaction, as neutralization occurred during the aqueous precipitation step. The yield of purified product was 1.97 g (98%). To ensure reproducibility, this propargylation procedure was repeated three times under identical conditions. The yields obtained in all three runs were highly consistent (average yield = 98 ± 1%).

#### 2.4.2 Lignin silanization

Hydrosilylation of propargylated lignin was carried out according to a modified version of Dobrynin *et al.*.^40^ In a typical reaction, 1.00 g of Prop-lignin was dispersed in 30 mL of chloroform and stirred at 70 °C for 1 hour to ensure full solubilization. Then, 275 mg of dimethylphenylsilane (Me_2_PhSiH) or 1,1,3,3-tetramethyldisiloxane (DH) was added, followed by 3 μL of Karstedt's catalyst. The reaction mixture was kept at 70 °C for 24 hours under an inert atmosphere to avoid oxidative side reactions. Upon cooling, the reaction mixture was filtered, and the product was precipitated by the slow addition of 300 mL of ethanol under vigorous stirring. The crude precipitate was then washed three times with a 7 : 3 ethanol–water mixture and once with diethyl ether to remove residual solvents and catalyst. To remove any trace metal ions and small-molecule impurities, the solid was subjected to dialysis for 48 hours against an aqueous solution of disodium EDTA (0.01 M), followed by a final rinse with distilled water. The purified product was vacuum-dried at 50 °C for 24 hours. This hydrosilylation protocol was also validated for reproducibility. Triplicate reactions under the same conditions yielded consistent product masses (see supplementary Table S1).

### 2.5 Preparation of resins and plywood panels

#### 2.5.1 Formulation of resins

Resin was synthesized following the method outlined by Moubarik *et al.*.^41,42^ A phenol-formaldehyde (PF) resol resin with a viscosity of around 450 cP and 46 wt% solids content was synthesized by reacting formaldehyde with phenol in a 2.2 : 1 molar ratio, using sodium hydroxide (7.3 wt%) as a catalyst. The reaction was carried out in a 2 L glass reactor with temperature control and mechanical stirring. Reagents were added based on the formulation, and the reaction's progress was monitored at 90 °C by measuring the viscosity at 25 °C. The adhesives were prepared through the copolymerization of lignin and silylated lignin (in different proportions with PF resin) at room temperature. Four distinct weight ratios of lignin and silylated lignin to PF resin were selected for the formulations: 5 : 95, 7 : 93, 10 : 90, and 15 : 85 (w : w). Following this, the adhesives were kept at room temperature.

#### 2.5.2 Resins properties

Bonding performance of resins containing different lignin and silylated lignin contents was evaluated for joint strength using an Instron Testometric M500-50 AT machine, with a crosshead speed of 1 mm min^−1^. Maritime pine (*Pinus pinaster*) veneer was sliced into rectangular sections (2.5 cm × 11.5 cm × 3 mm), and resin was applied to a 2.5 cm × 2.5 cm area on one end of each piece, using a spread rate of 120 g m^−2^ (dry weight basis). To calculate the resin's solid content (%), 1 g of PF resin was dried in an aluminum dish at 100 °C for 4 hours, then calculating the ratio of dry weight to initial weight, multiplied by 100. The average solid content was calculated based on triplicate measurements. Gelation time was measured in triplicate for each adhesive by placing the mixture in a test tube, which was then immersed in boiling water. The adhesive was stirred with a wire spring until gel formation, and the time from immersion to gelation was recorded using a stopwatch. Viscosity of the resins was measured at 25 °C using a rotary rheometer (RheolabQC Anton Paar).

#### 2.5.3 Plywood manufacturing

The veneers of maritime pine (*Pinus pinaster*) had a moisture content of 4% and a thickness of 2 mm were used to create five-layer plywood panels that measured 250 mm × 250 mm × 10 mm. Adhesives were applied with brushes to both sides of the veneer at a spread rate of 120–130 g m^−2^ (dry weight). Each veneer, coated with adhesive, was stacked between two uncoated veneers, positioned perpendicular to one another. The assembled plywood was hot-pressed at 12 bar pressure for 6 minutes at 130 °C to replicate industrial gluing conditions and ensure complete curing of the adhesive.^41,42^ The mechanical properties of the plywood were evaluated, including modulus of rupture (MOR) and modulus of elasticity (MOE) in dry static bending, measured according to EN 310, and shear strength (SS), determined per EN 314. Each test was conducted on 50 specimens.

#### 2.5.4 Formaldehyde emission

The plywood's formaldehyde emissions were assessed in accordance with ISO/CD 12460-4, a European standard. The procedure utilized a 10 L glass desiccator containing five plywood samples (15 cm × 5 cm × 1 cm). Formaldehyde released by the samples was quantified using a photometric method. Each adhesive formulation was tested in triplicate.^21,41^

## 3 Results and discussion

### 3.1 Characterization of unmodified and modified lignins

#### 3.1.1 Fourier transform infrared spectroscopy

**Fig. 1:**
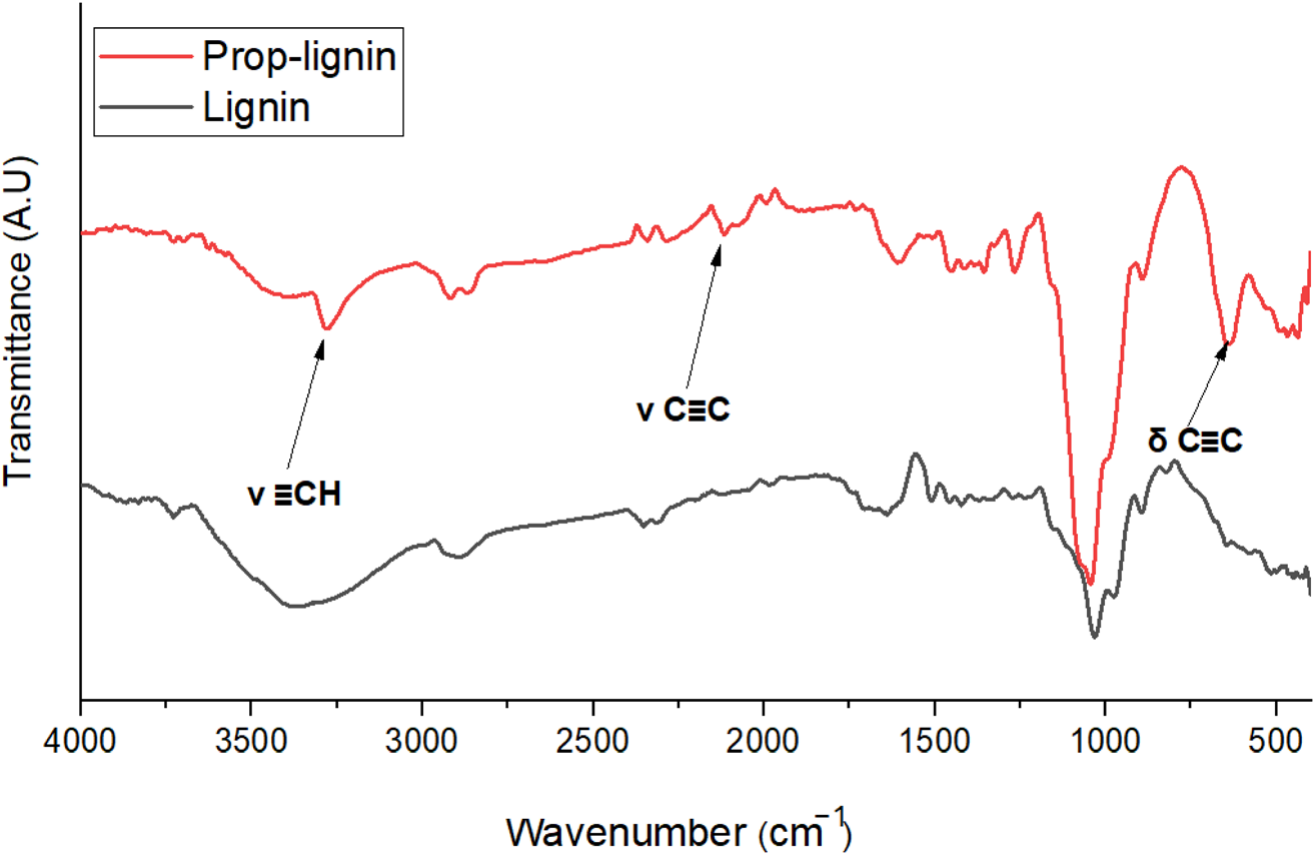
FTIR spectrum of the propargylated lignin in comparison with spectrum of lignin. (Band positions in cm^−1^).

FTIR spectral analysis (Fig. 1) was used to confirm successful propargylation by monitoring the appearance of characteristic bands for propargyl groups: 3280 cm^−1^ for *ν*(≡C–H), 2115 cm^−1^ for *ν*(C≡C), and 636 cm^−1^ for *δ*(C≡C). Following propargylation, a significant decrease in the broad –OH stretching band, initially dominant in the 3000–3670 cm^−1^ region,^43^ was observed. This reduction indicates the consumption of hydroxyl groups, confirming the successful grafting of propargyl moieties onto lignin. These results are consistent with previous studies by Wang *et al.*^44^ and Sen *et al.*^45^

**Fig. 2:**
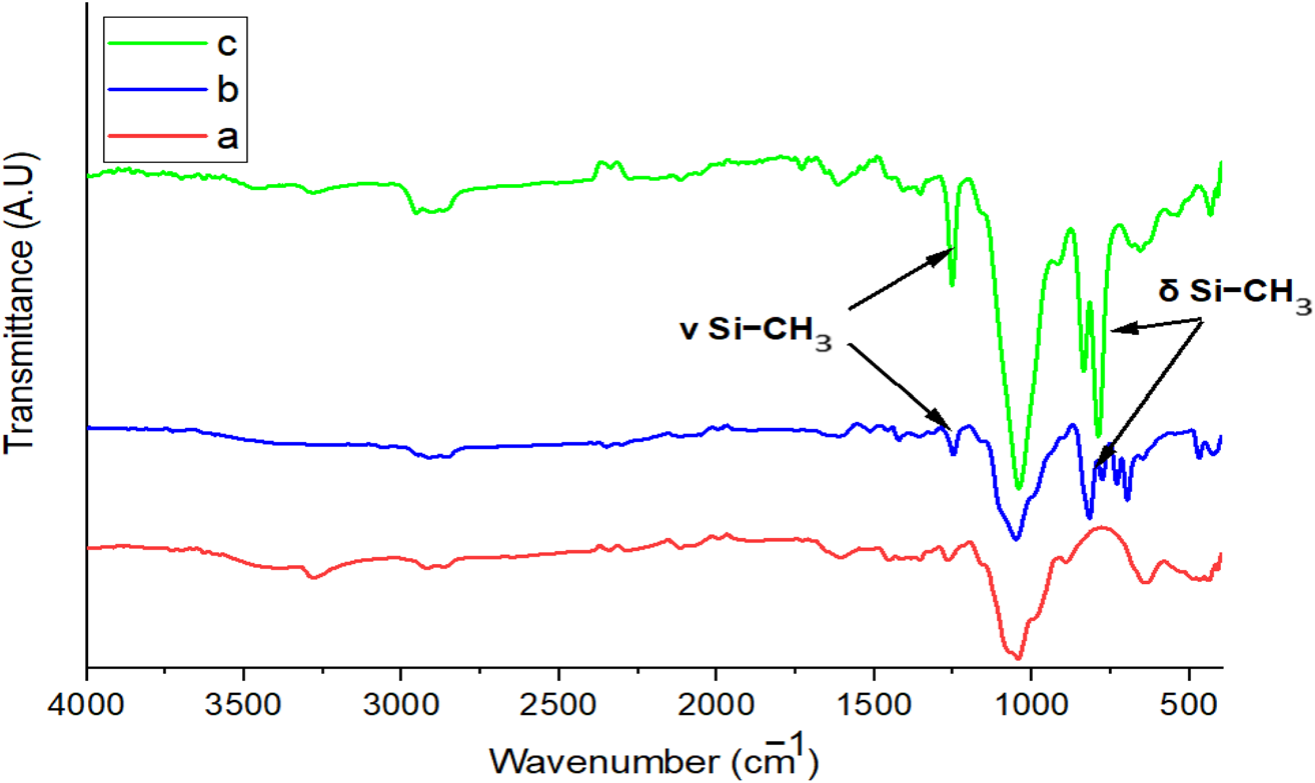
FTIR spectrum of (a) Prop-lignin, (b) Prop-lignin-Me_2_PhSiH, and (c) Prop-lignin-DH.

Hydrosilylation of propargylated lignin was confirmed by FTIR (Fig. 2 and Table 1). The disappearance of the propargyl-related bands (*ν*(C≡C), *ν*(≡C–H), and *δ*(C≡C)) together with the Si–H stretching band (2100–2250 cm^−1^) confirmed the successful reaction between propargyl groups and hydrosilanes.^46^

**Table 1:** Characterization of the resulting products

Reaction	Hydrosilane used	Formula of hydrosilane	Y%	% Si	FTIR band positions in cm^−1^
b	Dimethylphenylsilane Me_2_PhSiH	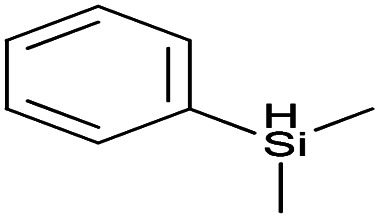	76.5	1.77	*ν* Si–CH_3_ (1248)
					*ν* Si–Ph (1110–1430)
					*δ* Si–CH_3_ (819)
c	1,1,3,3-Tetramethyldisiloxane DH	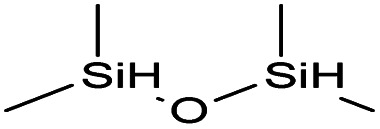	95	9.44	*ν* Si–CH_3_ (1254)
					*ν* Si–O–Si (1255)
					*δ* Si–CH_3_ (837)

#### 3.1.2 Thermogravimetric analysis (TG/DTG)

**Fig. 3:**
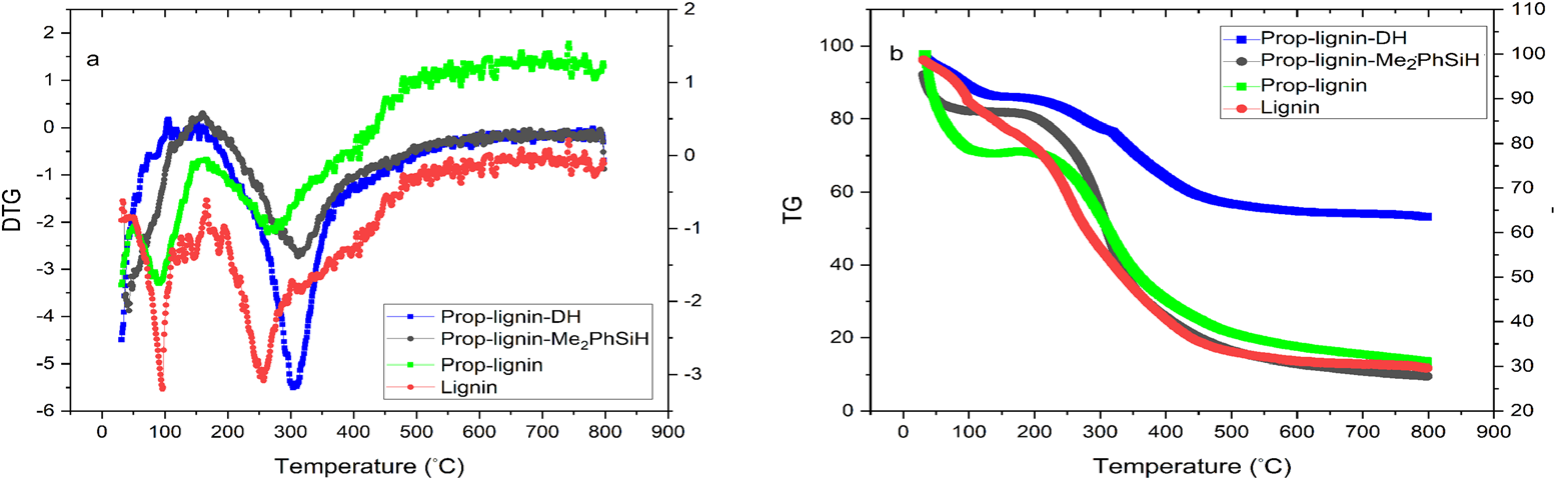
(a) DTG and (b) TG curves for lignin and its derivatives.

The thermal stability of lignin and its modified derivatives was assessed using TGA and DTG (Fig. 3). Two main events were identified: water evaporation below 150 °C and thermal decomposition of lignin. The onset decomposition temperatures (*T*_onset_) for lignin, Prop-lignin, Prop-lignin-Me_2_PhSiH, and Prop-lignin-DH were 158, 160, 166, and 170 °C, respectively. The maximum degradation temperatures (*T*_max_) were 256, 271, 313, and 309 °C, respectively.

In addition, the residual mass at 600 °C increased from 11.38% for unmodified lignin to 43.10% for Prop-lignin-DH, indicating improved thermal stability upon modification. This unusually high char yield is attributed to the incorporation of silane–siloxane moieties, which favor the formation of thermally stable Si–O–Si crosslinked networks. These inorganic domains act as a protective barrier during heating, thereby limiting the molecular degradation of the organic backbone.^47^ These results suggest that silane–siloxane functionalities not only enhance lignin's thermal resistance but also contribute to the accumulation of inorganic residues, explaining the elevated char fraction. Thus, the incorporation of silylated lignin significantly improves its potential as a thermally stable reinforcing agent in bio-composites.

#### 3.1.3 Scanning electron microscopy (SEM)

**Fig. 4:**
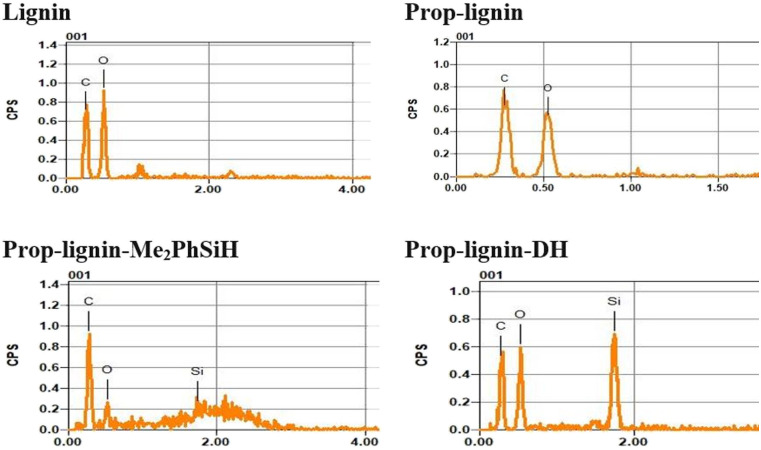
EDX spectra and corresponding elemental composition analysis.

The surface composition of the crosslinked lignin compounds was determined using EDX and SEM analysis. In each sample, oxygen (O), carbon (C), and silicon (Si) were identified (Fig. 4). Table 2 presents the quantitative surface composition. The carbon content increased from 43.88% (lignin) to 55.98% (Prop-lignin), confirming the successful attachment of propargyl groups.

**Table 2:** The spatial distribution of elements in lignin and its derivatives by SEM-EDX quantification

Element	Lignin		Prop-lignin		Prop-lignin-Me_2_PhSiH		Prop-lignin-DH	
	Weight %	Atom %	Weight %	Atom %	Weight %	Atom %	Weight %	Atom %
C	43.88	51.02	55.98	62.88	65.95	72.52	49.26	58.43
O	56.12	48.98	44.02	37.12	32.27	26.64	41.30	36.78
Si	0	0	0	0	1.77	0.83	9.44	4.79
Total	100	100	100	100	100	100	100	100

Silicon, absent in unmodified lignin and Prop-lignin, was detected at 1.77 wt% in Prop-lignin-Me_2_PhSiH and 9.44 wt% in Prop-lignin-DH, confirming successful hydrosilylation.

**Fig. 5:**
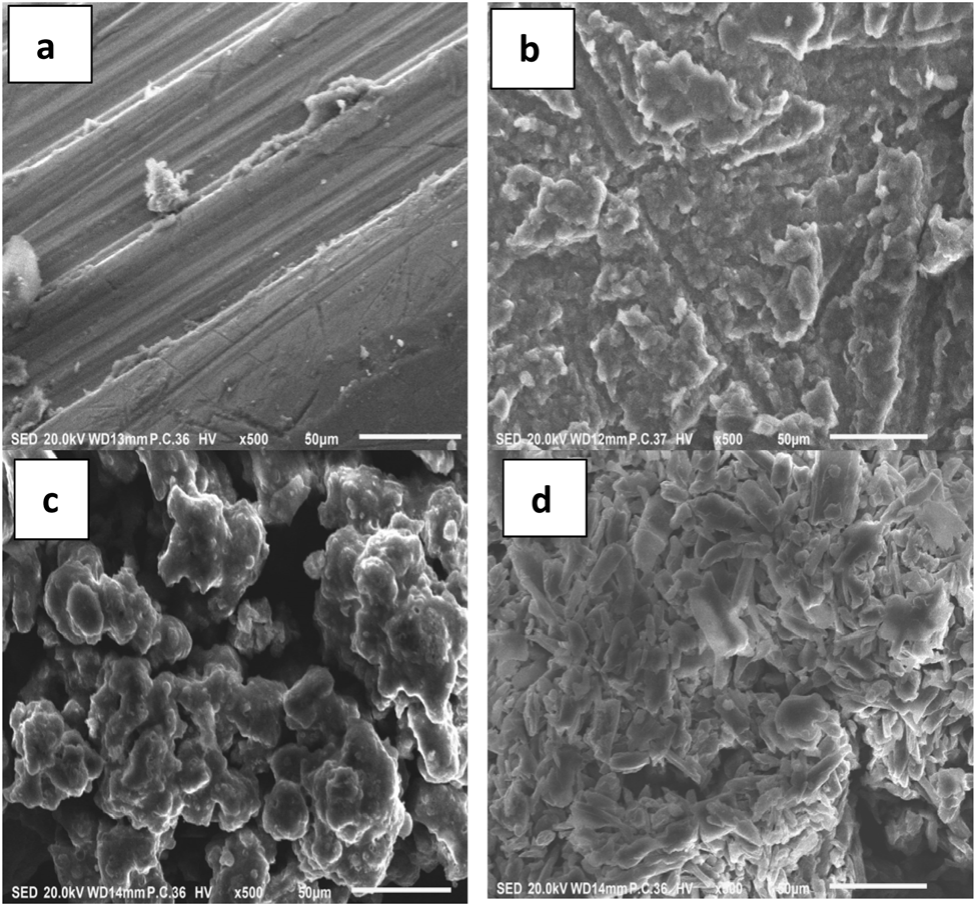
SEM micrographs of (a) lignin, (b) Prop-lignin, (c) Prop-lignin-Me_2_PhSiH and (d) Prop-lignin-DH.

SEM images (Fig. 5) revealed clear morphological differences between unmodified and modified lignin. Prop-lignin displayed a less uniform structure with finer surface features, indicating chemical modification. After hydrosilylation, the lignin surface exhibited embedded white clusters attributed to silane domains, which increased in density depending on the silane type. Modified lignins exhibited smoother surfaces than neat lignin, which is beneficial for enhancing compatibility with polymer matrices and improving adhesive properties.

#### 3.1.4 Solid-state nuclear magnetic resonance spectroscopy

**Fig. 6:**
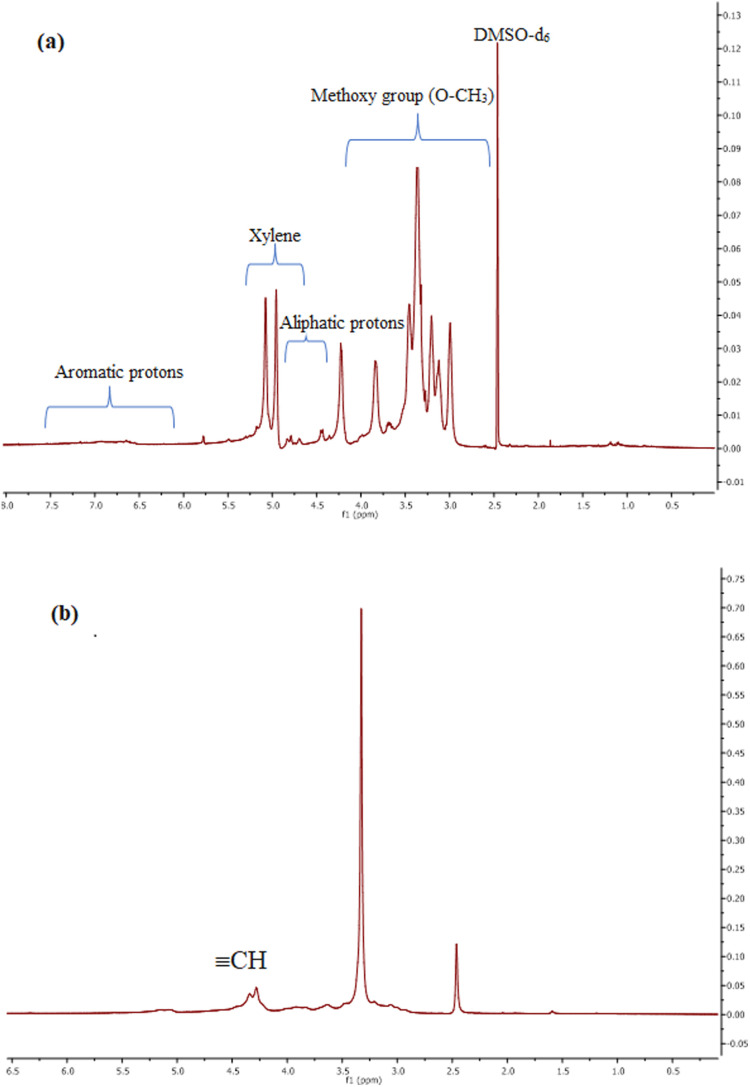
^1^H NMR spectrum of lignin (a) and propargylated lignin (b).

Fig. 6(a) shows the ^1^H NMR spectrum of isolated lignin, with assignments based on previous literature.^48–50^ A peak at 1.85 ppm corresponds to aliphatic hydrogens; signals at 2.46 ppm arise from DMSO-*d*_6_. Peaks between 3.00 and 4.23 ppm indicate methoxy groups (O–CH_3_), while those at 4.34 and 4.85 ppm represent aliphatic protons. Minor peaks from 4.96 to 5.08 ppm likely stem from carbohydrate contaminants. Aromatic proton signals appear between 6.00–8.00 ppm.

The broad signal in the 4.3–4.9 ppm region in Fig. 6(b) corresponds to alkyne protons, confirming successful propargylation. This observation is consistent with Sen *et al.*.^45^

#### 3.1.5 Water contact angle measurements

Lignin and silane–siloxane derivatives are inherently immiscible due to their contrasting polarities. However, chemical crosslinking—particularly through covalent Si–C bonds formed during hydrosilylation—enables their integration into a common matrix, enhancing the surface properties of lignin. This modification is particularly relevant for improving water resistance, since lignin's native hydroxyl (–OH), methoxy (–OCH_3_), and carbonyl (–C=O) groups contribute to high wettability.

**Fig. 7:**
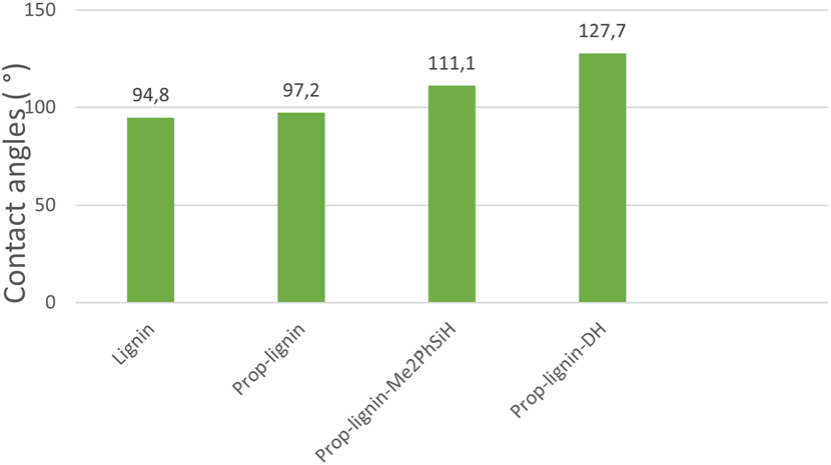
Main contact angle data for the prepared samples.

WCA measurements (Fig. 7) support this mechanistic interpretation. Unmodified lignin exhibited a contact angle of 94.8°, reflecting its polar surface. After propargylation with propargyl bromide, the WCA increased to 97.2°, indicating a reduction in polarity likely due to partial substitution of hydroxyl groups by hydrophobic alkyne moieties, which limit hydrogen bonding with water.

Hydrosilylation further amplified this effect. Prop-lignin modified with Me_2_PhSiH showed a WCA of 111.1°, demonstrating enhanced hydrophobicity due to the presence of nonpolar Si–C and Si–CH_3_ bonds. Notably, Prop-lignin-DH achieved a WCA of 127.7°, the highest among all samples. This is attributed to the flexible Si–O–Si siloxane backbone and the dense distribution of methyl groups, which increase surface hydrophobicity and steric hindrance to water adsorption.

These progressive increases in WCA correlate with the structural changes introduced at each modification step, validating the role of Si–C linkages and surface methylation in enhancing water repellency. Consequently, these chemically modified lignins are promising candidates for applications demanding strong moisture resistance, such as coatings and adhesives.

#### 3.1.6 Solubility measurements

**Fig. 8:**
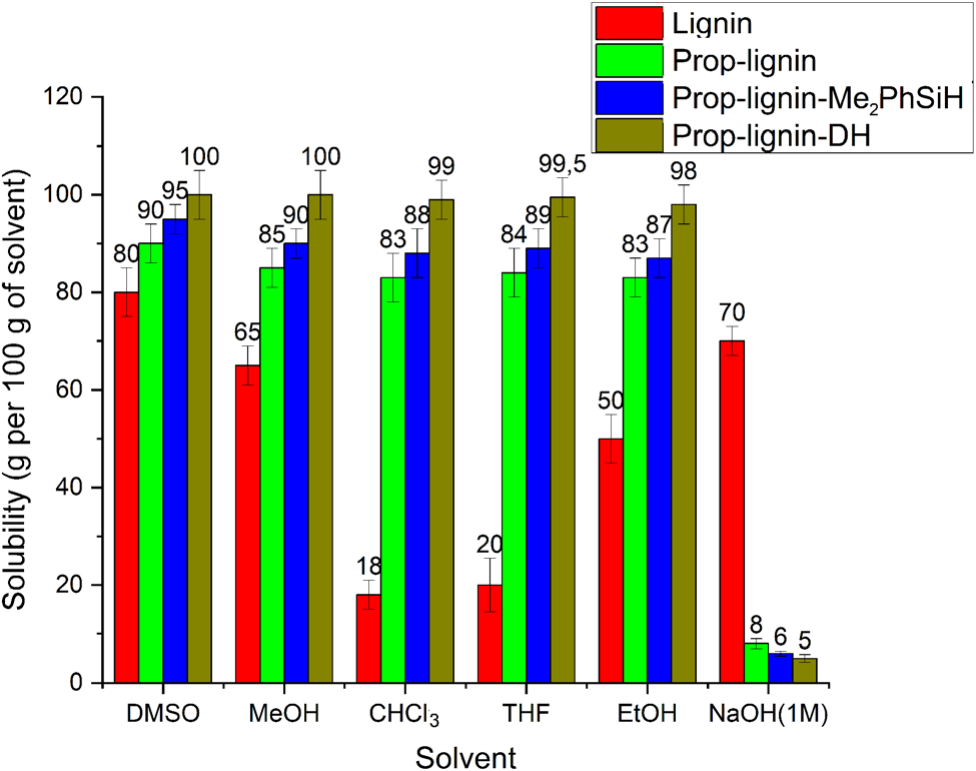
Solubility diagram of lignin and modified lignin in various solvents at 25 °C.

Fig. 8 compares the solubility of unmodified and modified lignins in several organic solvents and 1 M NaOH at 25 °C. DMSO proved the most effective solvent for all lignins, due to its high polarity and aprotic nature (dielectric constant ∼100). Modified lignins showed reduced solubility in NaOH. Additionally, the results demonstrated that unmodified lignin is nearly insoluble in chloroform and THF, Prop-lignin-DH is more soluble than Prop-lignin-Me_2_PhSiH and Prop-lignin in both THF and chloroform. Despite lignin's solubility parameter being closer to that of ethanol than methanol, methanol proves to be a better solvent for lignin. This can be explained by methanol's smaller molar volume compared to ethanol. The dissolution rates are significantly influenced by the molar volume of the solvents, with smaller solvents leading to higher penetration rates.

The observed decrease in solubility in polar solvents (*e.g.*, NaOH) for modified lignins is directly correlated with the introduction of hydrophobic groups and the formation of covalent Si–C and Si–O–Si bonds during the dual-functionalization process. These structural changes significantly reduce the number of hydroxyl groups available for hydrogen bonding with polar solvents, thereby increasing hydrophobicity and altering solvation behavior.

Notably, Prop-lignin-DH demonstrated the highest solubility, consistent with its higher surface hydrophobicity measured by WCA (127.7°). This can be attributed to its flexible siloxane (Si–O–Si) backbone and high surface density of methyl groups, which enhance compatibility with less polar environments.

These solubility trends confirm the mechanistic role of dual-functionalization in modifying the lignin surface, making it more hydrophobic and suitable for applications such as adhesives. This is also crucial for processability at an industrial scale, where solvent choice and solubility behavior directly influence blending with polymers, curing efficiency, and long-term stability of the final material.

### 3.2 Characterization of resins and plywood panels

#### 3.2.1 Formulated resins characterization

Table 3 presents the pH, solid content, gel time at 120 °C, and viscosity of the control PF, Lignin : PF, and Prop-lignin-DH : PF adhesives with different weight ratios. The pH of the neat PF resins remained stable and unchanged upon the incorporation of lignin and Prop-lignin-DH, even at higher concentrations. The solid content increased with increasing loading ratios of lignin and Prop-lignin-DH, reaching peak values of 59% and 60%, respectively, at the 15 : 85 ratio. In contrast, the control PF resin had a solids content of 43%. A notable decrease in gel time was also observed as the proportion of these fillers increased. Specifically, the gel time decreased from 966 s (PF alone) to 677 s for lignin and to 661 seconds for Prop-lignin-DH. This indicates that the crosslinking process occurs significantly faster, resulting in a higher degree of networking between lignin or Prop-lignin-DH and phenol to form lignin-PF and Prop-lignin-DH : PF condensates. The enhanced reactivity of Prop-lignin-DH compared to unmodified lignin highlights the effectiveness of the chemical modification, which increases the availability of reactive sites on the lignin molecule, thereby improving its compatibility with phenol-formaldehyde and accelerating the gelation process. On the other hand, viscosity increased linearly, reaching a maximum in the formulation with up to 15% of both resins lignin : PF (752 cP), Prop-lignin-DH : PF (721 cP) compared to the pure PF formulation (417 cP), the increase in resin viscosity with lignin and Prop-lignin-DH addition is due to higher solids content and enhanced cross-linking (cohesion effect). These findings are consistent with the previously developed lignin-based PF resins.^21,34,36^ In addition, all the adhesives formulated in this study comply with the Chinese standard for wood adhesives (GB/T 14732), demonstrating their suitability for wood bonding applications.

**Table 3:** Resin physicochemical characteristics at different loading rates

Adhesives		pH	Solid content, (%)	Gel time at 120 °C, (s)	Viscosity, (cP)
**Control PF**	0//100	11.20	43	966	417
**Lignin//PF**	5//95	11.18	47	817	492
	7//93	11.17	51	774	524
	10//90	11.15	54	710	579
	15//85	11.14	59	677	752
**Prop-lignin-DH//PF**	5//95	11.21	49	829	490
	7//93	11.19	52	781	554
	10//90	11.17	54	695	639
	15//85	11.16	60	661	721

#### 3.2.2 Resin bond strength and wood failure

**Fig. 9:**
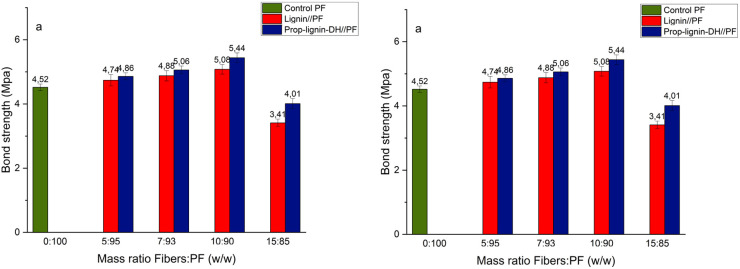
Bond strength (a) and wood failure (b) of plywood with two layers, bonded using control PF resin, lignin-PF resin, and Prop-lignin-DH resin at different loading ratios.

Optimizing and characterizing the resin properties is crucial to creating a novel wood adhesive. Bond strength and wood failure percentages are critical parameters that reflect the adhesive's quality. These properties were assessed for various formulations and benchmarked against the control PF resin. The results, presented in Fig. 9, show the impact of lignin and dual-functionalized lignin (Prop-lignin-DH) substitution at different weight ratios on adhesive performance, helping to identify the optimum formulation with better bonding and wood failure characteristics.

Bond strength and wood failure were determined in triplicate (*n* = 3) for each formulation. Statistical significance of differences between the control and modified resins was assessed using one-way ANOVA at a 5% significance level (*p* < 0.05).

The control PF resin (0 : 100) exhibited a bond strength of 4.52 ± 0.10 MPa and 80% wood failure. Upon substitution with 10 wt% lignin, the bond strength increased to 5.08 ± 0.12 MPa, accompanied by 90% wood failure. In contrast, substitution with 10 wt% Prop-lignin-DH resulted in a further increase, yielding a bond strength of 5.44 ± 0.09 MPa and 100% wood failure. Both improvements were statistically significant compared to the control (*p* < 0.05).

However, increasing the substitution level to 15 wt% led to a marked decline in adhesive performance: the bond strength dropped to 3.41 ± 0.20 MPa for lignin and 4.01 ± 0.15 MPa for Prop-lignin-DH (*p* < 0.01). This reduction is attributed to micellization and aggregation phenomena of lignin-derived macromolecules during polymerization, which hinder effective cross-linking and network formation.

A similar pattern was observed in the wood failure data: at 15 wt% substitution, wood failure decreased significantly to 65% (lignin) and 70% (Prop-lignin-DH) (*p* < 0.01), further confirming that excessive incorporation of lignin derivatives negatively affects adhesive performance.^21,36^

Importantly, the panels containing Prop-lignin-DH consistently outperformed those with unmodified lignin at equivalent loading. This improvement is attributed to the dual functionalization of lignin with propargyl and hydrosilyl groups. These moieties enhance lignin's reactivity by introducing new cross-linkable sites: the propargyl groups enable click-like or nucleophilic additions, while the hydrosilyl groups undergo condensation and siloxane bridge formation during curing. This synergistic dual reactivity improves compatibility with phenol and formaldehyde and reinforces the resin's polymeric network, resulting in higher cohesive strength and interfacial adhesion.

Previous studies have reported similar trends regarding the incorporation of lignin into phenol-formaldehyde (PF) resins; however, This system demonstrates superior performance at lower substitution levels. For example, Mennani *et al.* achieved 5.24 MPa and 95% wood failure using 20 wt% lignin,^21^ while in the current study, 5.44 MPa and 100% wood failure were achieved with only 10 wt% of Prop-lignin-DH. Younesi-Kordkheili *et al.* reached 100% wood failure with 70% DES-pretreated nanolignin but reported only 2.3 MPa bond strength.^36^ Similarly, Solt *et al.* obtained 6.0 MPa with 50% kraft lignin, but at the cost of excessive lignin usage.^51^ Zhao *et al.* reported 1.11 MPa and 93% wood failure at 40% phenolated lignin content, with significant performance drops at higher loadings.^52^

In contrast, the current formulation using only 10 wt% dual-functionalized lignin not only achieves the highest wood failure (100%) but also one of the highest bond strengths (5.44 MPa) reported among comparable bio-based systems.

This confirms that the proposed dual modification strategy confers significant chemical and mechanical advantages over unmodified lignin systems. The combination of propargyl and hydrosilane functionalities promotes optimal integration of lignin into the PF resin matrix, improving both reactivity and performance at minimal substitution levels. Thus, this strategy represents a more efficient and scalable approach for developing high-performance, lignin-based phenolic adhesives.

#### 3.2.3 Panel mechanical properties

The optimal incorporation ratios of lignin and Prop-lignin-DH were selected to evaluate their effectiveness as bio-based binders and to assess their impact on the mechanical performance of the resulting wood panels. Table 4 summarizes the key performance indicators, including dry modulus of rupture (MOR), dry modulus of elasticity (MOE), shear strength under dry, cold water, and boiling water conditions, as well as formaldehyde emissions (FE) from the manufactured composites.

**Table 4:** SS, MOR, MOE and FE of plywood panels glued with control PF, optimal resins lignin-PF and Prop-lignin-DH-PF

Plywood panels	Shear strength mean ± SD (N mm^−2^)					MOR mean ± SD (N mm^−2^)	MOE mean ± SD (N mm^−2^)	Formaldehyde emission mean ± SD (mg m^−2^ h^−1^)
	Dry	Cold water at 20 °C for 24 h	Boiling water after					
			3 h	6 h	9 h			
Control PF (0 : 100)	1.80 ± 0.08	1.71 ± 0.08	1.52 ± 0.10	0.97 ± 0.14	0.54 ± 0.07	48 ± 2.09	3258 ± 75	2.51 ± 0.26
Lignin : PF 10 : 90 (w/w)	2.04 ± 0.15	1.82 ± 0.10	1.51 ± 0.08	1.21 ± 0.12	0.80 ± 0.07	57 ± 1.39	3605 ± 83	2.31 ± 0.12
Prop-lignin-DH : PF 10 : 90 (w/w)	2.81 ± 0.11	2.01 ± 0.09	1.75 ± 0.07	1.49 ± 0.10	0.94 ± 0.08	66 ± 1.02	4148 ± 81	2.01 ± 0.13
GB/T 17657-2013	>0.7	> 0.7						
EN 16352	>1							
EN310						20.2	2280	
EN13986 : 200 E1								≤3.5
EN13986 : 2005 E2								≤8

The initial results showed that the reference panels produced with the control PF resin exhibited a modulus of elasticity (MOE) of 3258 ± 75 N mm^−2^ and a modulus of rupture (MOR) of 48 ± 2.09 N mm^−2^, establishing the baseline mechanical performance of the unmodified adhesive. The incorporation of 10% lignin or Prop-lignin-DH into the PF resin during polymerization significantly improved the mechanical properties of the panels. Specifically, the MOE increased by 10.7% and 27.3%, while the MOR improved by 19% and 38%, respectively, compared to the reference formulation. ANOVA analysis confirmed that these enhancements were statistically significant (*p* < 0.05), particularly in the case of the Prop-lignin-DH-modified resin, underscoring the superior reinforcing potential of the functionalized lignin derivative.

The observed enhancement in mechanical performance is primarily attributed to the chemical interactions occurring between the hydroxymethyl groups of the PF resin and the phenolic hydroxyl groups present in lignin during polymerization. These interactions facilitate the formation of a densely cross-linked network, leading to significant improvements in both MOR and MOE compared to the unmodified PF resin. However, even greater enhancements were achieved with the incorporation of Prop-lignin-DH—a lignin derivative functionalized through propargylation and subsequent hydrosilylation. This dual modification increases the reactivity and compatibility of lignin with the PF matrix, particularly through the formation of siloxane (Si–O) bonds between the hydrosilyl groups and lignin hydroxyls.^16,19^ These Si–O bonds act as robust chemical bridges between lignin and the resin matrix, reinforcing the three-dimensional polymer network during curing.^28,53^ The resulting structure is more densely cross-linked, which improves both the stiffness (MOE) and strength (MOR) of the adhesive. This denser molecular architecture enhances the rigidity of the composite panels, making them more resistant to mechanical deformation and internal failure. The significantly higher MOE and MOR values observed in panels with Prop-lignin-DH highlight the superior mechanical reinforcement achieved through this functionalization strategy, outperforming those obtained with unmodified lignin.

Numerous studies have confirmed that incorporating lignin into phenolic adhesives enhances their mechanical performance compared to fully petrochemical-based PF resins. In parallel, silicon-based compounds have also been shown to improve the properties of PF adhesives. However, to date, no studies have specifically addressed the use of silylated lignin in this context. For instance, Hafezi *et al.* investigated the incorporation of an aminosilane coupling agent into urea-formaldehyde adhesives for bonding particleboards made from wheat straw and poplar wood. Their results demonstrated that both the wheat straw-to-poplar ratio and the inclusion of silane significantly influenced board properties, with the addition of 10% silane notably improving the modulus of elasticity (MOE) and modulus of rupture (MOR).^54^

In another study, Mennani *et al.* reported that the optimal formulation of PF adhesives was achieved with 20 wt% extracted lignins, leading to enhanced MOE values of approximately 3505, 3536, and 3515 N mm^−2^, and MOR values of 55, 55, and 56 N mm^−2^ for lignins derived from cactus waste seeds (CWS), spent coffee grounds (SC), and sugarcane bagasse (SCB), respectively.^21^ Similarly, Benhamou *et al.* found that PF resins incorporating 10% raw CWS lignin attained a MOE of 3501 N mm^−2^ and a MOR of 53 N mm^−2^, outperforming the control PF resin, which showed a MOE of 3170 N mm^−2^ and a MOR of 46 N/mm^2.^^55^ Karri *et al.* also demonstrated that the incorporation of 50% lignin into PF resins significantly improved plywood bonding performance, yielding a MOR of 82 MPa and a MOE of 7.65 GPa, compared to 78.5 MPa and 5.28 GPa, respectively, for the unmodified PF resin.^56^

Compared to these studies, the current work demonstrates superior mechanical performance, with the incorporation of only 10% Prop-lignin-DH leading to a MOE and MOR improvement of 27.3% and 38%, respectively, over the control, highlighting the efficiency of the dual propargylation-hydrosilylation modification strategy in enhancing the structural cohesion and rigidity of PF-based wood composites.

The shear strength (SS) of the reference panels bonded with unmodified PF resin was found to be approximately 1.80 ± 0.08 N mm^−2^ in the dry state, 1.71 ± 0.08 N mm^−2^ after 24 hours in cold water at 20 °C, and 0.54 ± 0.07 N mm^−2^ after 9 h in boiling water, with the expected decrease in strength due to moisture exposure. In contrast, addition of 10% lignin significantly improved SS under all conditions, resulting in increases of 13.4% (dry), 6.5% (cold water), and 48.1% (boiling water). ANOVA confirmed that these increases were significant compared to pure PF panels.

Even more remarkable were the improvements observed with the incorporation of 10% Prop-lignin-DH, the lignin chemically modified *via* propargylation and hydrosilylation. This dual-functionalized lignin resulted in a 56.1% increase in SS under dry state, a 17.6% increase after cold-water exposure, and a 74.1% increase after 9 h in boiling water—highlighting the significant boost in moisture resistance and bonding integrity. ANOVA analysis further validated that the improvements associated with Prop-lignin-DH were statistically significant across all conditions.

The superior performance of Prop-lignin-DH is attributed to the enhanced compatibility and reactivity between the modified lignin and the PF resin matrix. The formation of stable siloxane (Si–O) bonds, resulting from hydrosilylation, strengthens the interfacial adhesion and reinforces the polymer network, thereby improving shear strength and overall durability under harsh environmental conditions. While the incorporation of unmodified lignin already contributes to improved mechanical performance by enhancing PF-lignin interactions, the chemical modification through propargylation and hydrosilylation enables the formation of a more robust and moisture-resistant crosslinked structure.

Additionally, as shown in Table 4, the plywood produced did not surpass the Chinese standard for wood adhesives (GB/T 17657-2013, 0.7 MPa), as well as the EN 16352 standard's minimum requirement of 1 MPa. However, the static bending performance exceeded the testing standards outlined in EN 310 (MOE > 2280 and MOR > 20.2).

Compared to earlier works, this study introduces a novel and highly effective strategy by using chemically modified lignin bearing siloxane functionalities. Previous studies have reported performance enhancements using either unmodified lignin or silane additives separately; however, none have explored the synergistic effect of dual modification *via* propargylation and hydrosilylation within a PF matrix. This approach not only overcomes one of the major limitations of bio-based phenolic resins—namely, poor moisture resistance—but also achieves significantly superior shear strength and dimensional stability. The findings establish Prop-lignin-DH as a promising candidate for the development of high-performance, bio-based wood adhesives that combine enhanced compatibility, mechanical robustness, and durability under challenging environmental conditions.^21,36,41,57^

#### 3.2.4 Emission of formaldehyde

A significant drawback associated with the use of phenol-formaldehyde (PF) resins in manufacturing wood-based materials lies in their formaldehyde emissions (FE). These emissions pose a substantial threat to public health and safety, as formaldehyde has been recognized as a carcinogenic substance by the U.S. Environmental Protection Agency (EPA) since 2008.^28,42^ This issue poses a critical challenge for the wood composite industry, which relies heavily on these resins for panel and product manufacturing.

The FE from cured resins during the lifespan of panels primarily result from the hydrolysis of chemical bonds within the resin and from the presence of residual free formaldehyde in the PF resin structure. In this study, we adopted an effective strategy to mitigate these emissions by incorporating 10% lignin and Prop-lignin-DH into PF resin formulations. As shown in Table 4, panels bonded with standard (control) PF resin exhibited high formaldehyde emission levels (2.51 ± 0.26 mg m^−2^ h^−1^). In contrast, incorporation of lignin and Prop-lignin-DH led to significant reductions in FE, with decreases of 8% and 20%, respectively, compared to the control. ANOVA confirmed the statistical significance of these reductions, which can be attributed to the partial substitution of PF resin by lignin derivatives during polymerization. In particular, the molecular structure of Prop-lignin-DH, enriched with reactive siloxane functionalities, enables it to capture formaldehyde *via* cross-linking interactions, thereby reducing overall toxicity.^58,59^

Phenolic groups represent the most critical functional moieties in lignin, as the phenylpropane units within them exhibit preferential reactivity toward formaldehyde in most chemical reactions involving this biomolecule.^21,58,60^ Functionalization with 1,1,3,3-tetramethyldisiloxane (DH) further enhances this reactivity by introducing siloxane groups, which not only improve compatibility with the PF matrix but also reinforce the network structure, leading to lower emissions. These findings underscore the potential of bio-based materials functionalized with silicon-containing compounds as effective strategies for reducing formaldehyde emissions in wood composites. Such materials represent eco-friendly alternatives and contribute to improving indoor air quality.

The incorporation of Prop-lignin-DH into PF resins offers multiple benefits, including enhanced mechanical properties and increased thermal stability, owing to the thermostable characteristics of siloxane groups,^53^ alongside a substantial reduction in formaldehyde release. While the use of lignin-based silicone additives in formaldehyde-rich systems is still emerging, this approach opens new prospects for research and innovation, potentially transforming current practices in the formulation of sustainable, high-performance adhesives.^58^

It is also noteworthy that the formaldehyde emission levels of the modified resins meet the EN 13986 : 2005 E1 (≤3.5 mg m^−2^ h^−1^) and E2 (≤8 mg m^−2^ h^−1^) standards, demonstrating that plywood produced with silylated lignin-based PF resins is suitable for both indoor and outdoor applications.^57^

The dual functionalization strategy applied to lignin offers significant advantages from both environmental and performance standpoints. The synthesis follows a green chemistry philosophy by operating under relatively mild conditions (*e.g.*, low to moderate temperatures, no strong acids or bases), and by valorizing lignin, an abundant and renewable by-product. Moreover, the method is compatible with industrial-scale processes due to its solvent-based flexibility and potential for batch or continuous production. Functionally, the introduction of both alkyne and silane groups allows for effective cross-linking within phenol-formaldehyde matrices, resulting in both improved mechanical strength and significant reduction in formaldehyde emissions—an important dual benefit for eco-friendly wood composites.

However, several limitations must be considered for industrial implementation. The use of functionalizing agents such as propargyl bromide and hydrosilanes can increase formulation costs and may raise safety or environmental concerns due to their reactivity and handling requirements. Although the reactions are efficient, ensuring consistent grafting and reactivity at scale may require further optimization of parameters such as mixing, catalyst loading, and reaction time. Additionally, while the approach enhances performance, the need for auxiliary curing agents or modified pressing cycles could impact process economics and energy consumption. Finally, the environmental footprint of the derivatization reagents must be weighed against the gains in performance and formaldehyde mitigation. Future work should therefore explore greener alternatives for functionalization and assess the full life-cycle impact of the modified lignin formulations.

## 4. Conclusions

Lignin was successfully dual-functionalized *via* propargylation and hydrosilylation, improving its compatibility and reactivity with phenol-formaldehyde (PF) resins. Incorporating up to 15 wt% lignin and Prop-lignin-DH into PF resins enhanced viscosity and solid content while reducing gelation time. The optimal formulation (10 wt% Prop-lignin-DH) yielded significant improvements in mechanical properties (MOR: +38%, MOE: +27.3%) and shear strength (up to +74.1%), together with a 20% decrease in formaldehyde emissions. These results demonstrate the potential of dual-functionalized lignin for developing high-performance, sustainable adhesives. Future work should focus on improving process scalability, minimizing reagent consumption, and broadening comparisons with commercial bio-based adhesives to better assess industrial relevance.

## Author contributions

Nadia ANTER: conceptualization, formal analysis, visualization, writing – original draft, writing – review & editing. Ahlam Chennani: visualization, conceptualization. Mohamed-Yassine Guida: conceptualization, formal analysis, visualization. Fatima ezzahra Atmani: formal analysis, visualization. Amine Moubarik: methodology, visualization. El Mostapha Rakib: methodology, resources. Abdelouahid Medaghri-Alaoui: supervision, visualization. Abdellah Hannioui: methodology, supervision, validation, visualization.

## Conflicts of interest

The authors declare that they have no known financial conflicts of interest or personal relationships that could have influenced the content of this article.

## Supplementary Material

RA-015-D5RA04233J-s001

## Data Availability

Supplementary information: FTIR, NMR, TGA/DTG, SEM-EDX spectra, contact angle, solubility profiles, and mechanical test results. See DOI: https://doi.org/10.1039/d5ra04233j.
