# Supplementary material for: Dual-functionalized lignin as a sustainable modifier for high-performance phenol-formaldehyde adhesives in plywood production
Source: RSC Adv. 2025 Sep 5;15(39):32118–30. doi: 10.1039/d5ra04233j (PMC12412671; doi:10.1039/d5ra04233j)
Supplement: RA-015-D5RA04233J-s001 [file RA-015-D5RA04233J-s001.pdf]

Supplementary Information (SI) for RSC Advances.
This journal is © The Royal Society of Chemistry 2025

Supporting Information

Title: Dual-functionalized lignin as a sustainable modifier for high-performance phenol-formaldehyde adhesives in plywood production

Authors: Nadia Anter [a*], Ahlam Chennani [a], Mohamed-Yassine Guida [a], Fatima ezzahra Atmani [b], Amine Moubarik [c], El Mostapha Rakib [a.d], Abdelouahid Medaghri-Alaoui [a.e], Abdellah Hannioui [a.e]

*Correspondence to: nadia.anter@usms.ma

Table des matières

1. Fourier transform infrared spectroscopy

2. Thermogravimetric analysis (TG/DTG)

3. SEM and EDX Results

4. Solid-state nuclear magnetic resonance spectroscopy

5. Water contact angle measurements

6. Solubility measurements

7. Formulated resins characterization

8. Resin bond strength and wood failure

9. Panel mechanical properties and Emission of formaldehyde

1. Fourier transform infrared spectroscopy

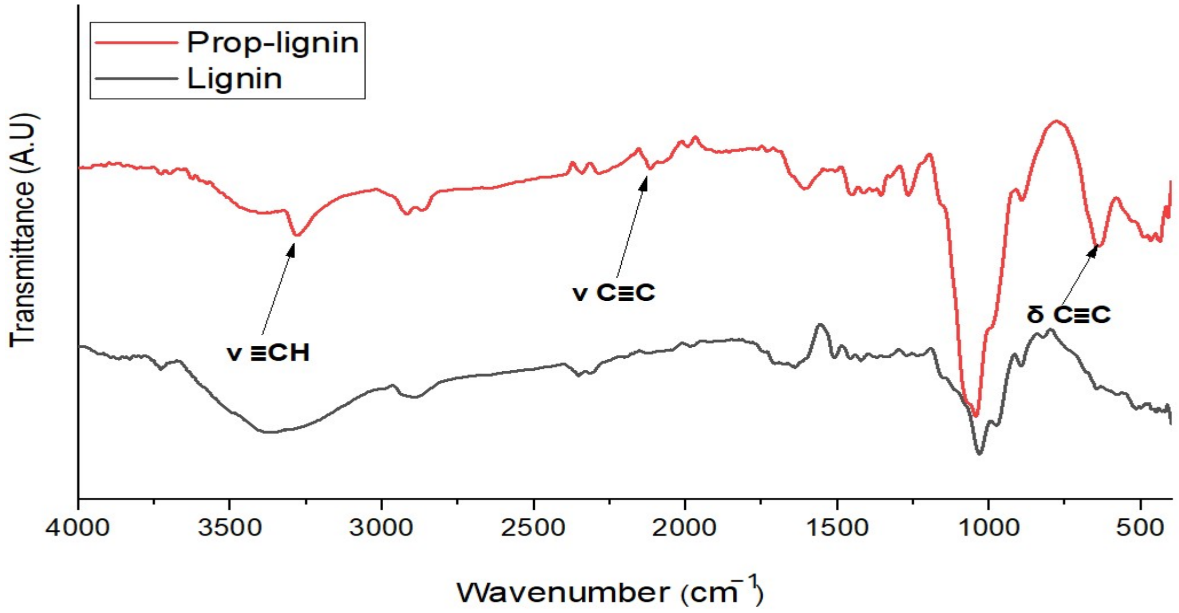

Fig. 1. FTIR spectrum of the propargylated lignin in comparison with spectrum of lignin. (Band positions in cm^{-1}).

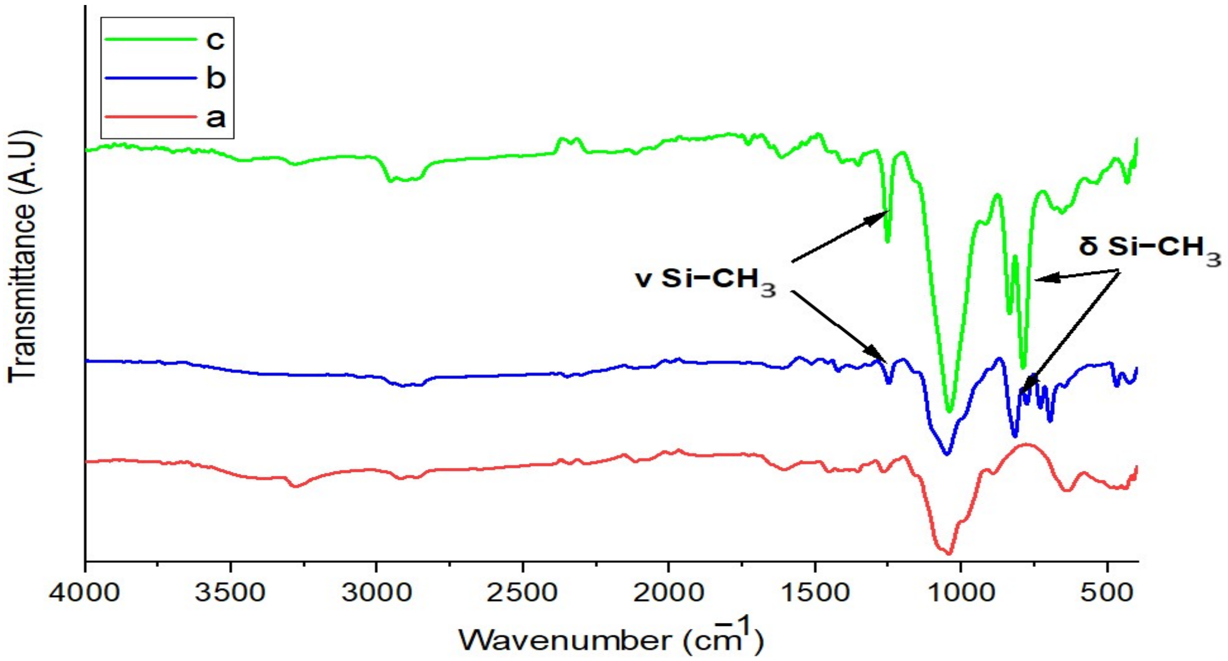

Fig. 2. FTIR spectrum of a) Prop-lignin, b) Prop-lignin-Me_2PhSiH, and c) Prop-lignin-DH.

Table 1. Characterization of the resulting products

Reaction	Hydrosilane used	Formula of hydrosilane	Y%	%Si	FTIR Band positions in cm^{-1}
**b**	Dimethylphenylsilane<br>Me_2PhSiH	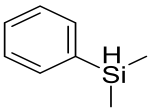	76.5	1.77	ν $Si-CH_3$ (1248)<br>ν Si−Ph (1110-1430)<br>δ $Si-CH_3$ (819)
**c**	1,1,3,3-tetramethyldisiloxane<br>DH	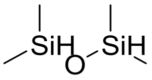	95	9.44	ν $Si-CH_3$ (1254)<br>ν Si−O−Si (1255)<br>δ $Si-CH_3$ (837)

2. Thermogravimetric analysis (TG/DTG)

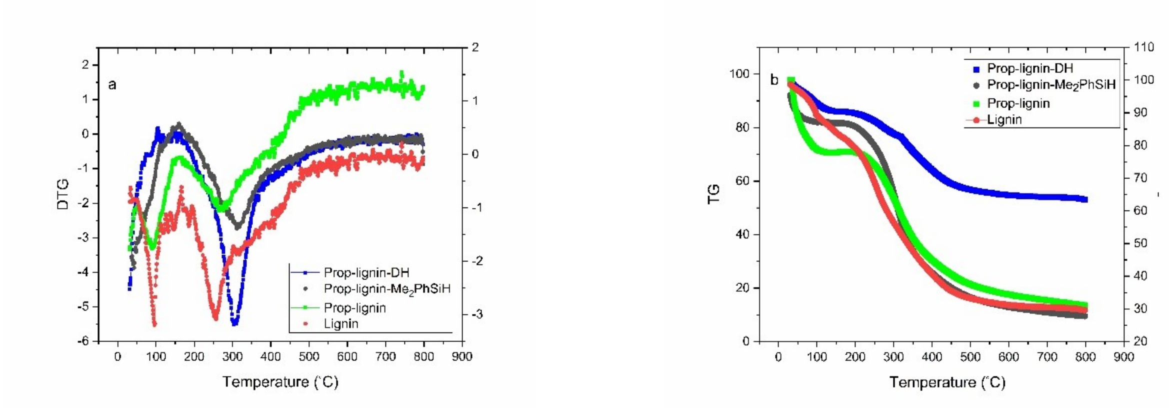

Fig. 3. (a) DTG and (b) TG curves for lignin and its derivatives.

3. SEM and EDX Results

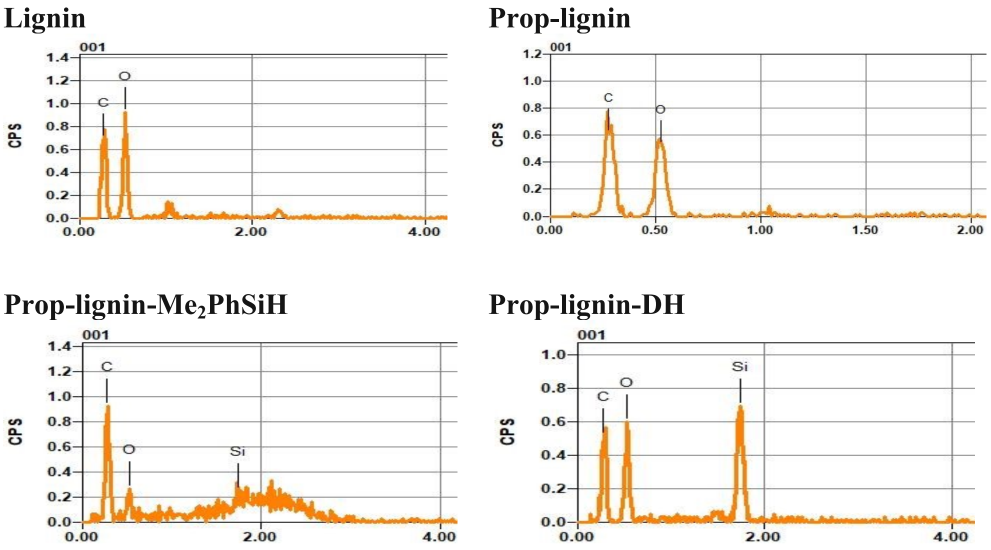

Fig. 4. EDX spectra and corresponding elemental composition analysis.

Table 2. The spatial distribution of elements in lignin and its derivatives by SEM-EDX quantification

Element	Lignin		Prop-lignin		Prop-lignin-Me_2PhSiH		Prop-lignin-DH	
	Weight%	Atom%	Weight%	Atom%	Weight%	Atom%	Weight%	Atom%
**C**	43.88	51.02	55.98	62.88	65.95	72.52	49.26	58.43
**O**	56.12	48.98	44.02	37.12	32.27	26.64	41.30	36.78
**Si**	0	0	0	0	1.77	0.83	9.44	4.79
**Total**	100	100	100	100	100	100	100	100

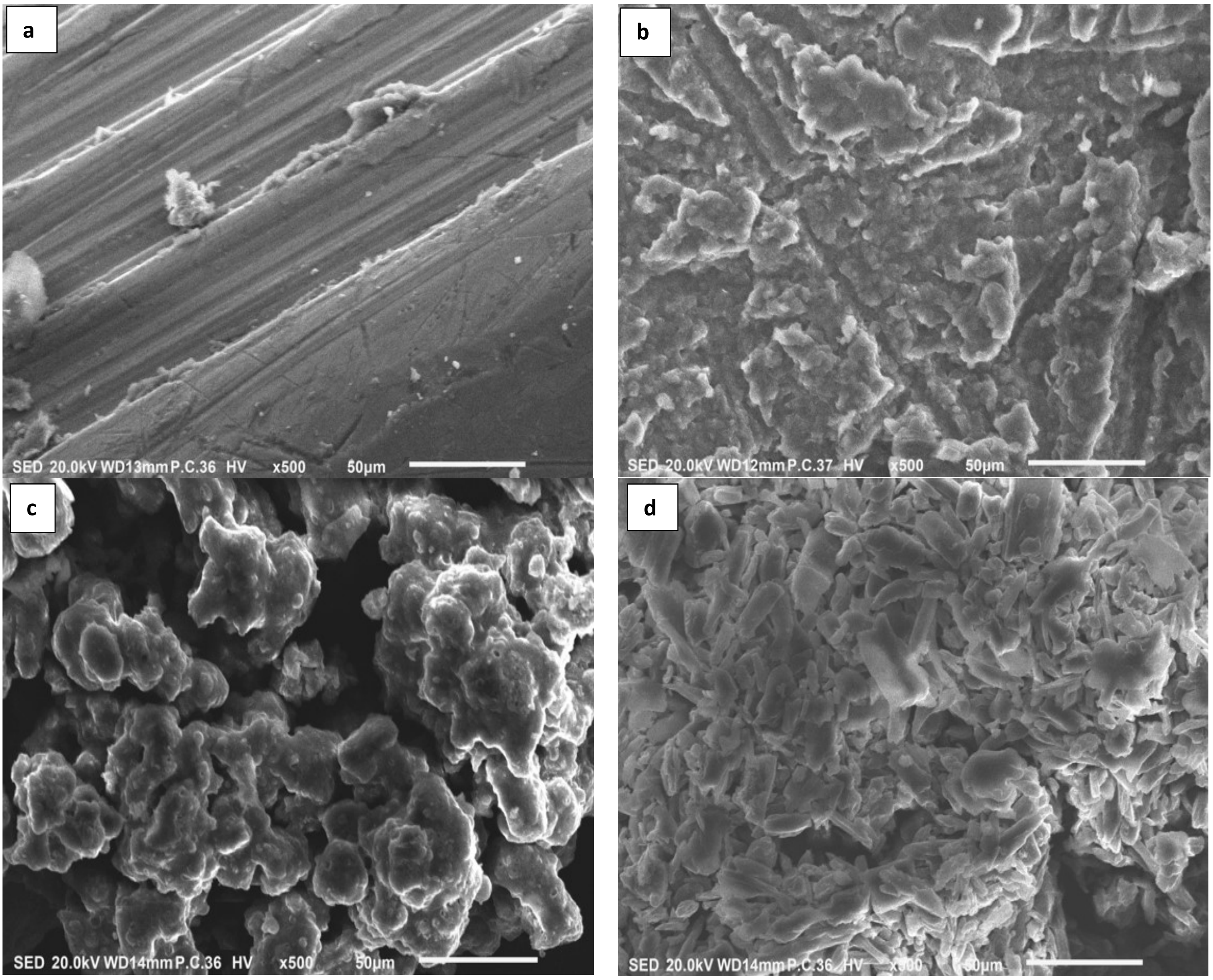

Fig. 5. SEM micrographs of a) lignin, b) Prop-lignin, c) Prop-lignin-Me_2PhSiH and d) Prop-lignin-DH.

4. Solid-state nuclear magnetic resonance spectroscopy

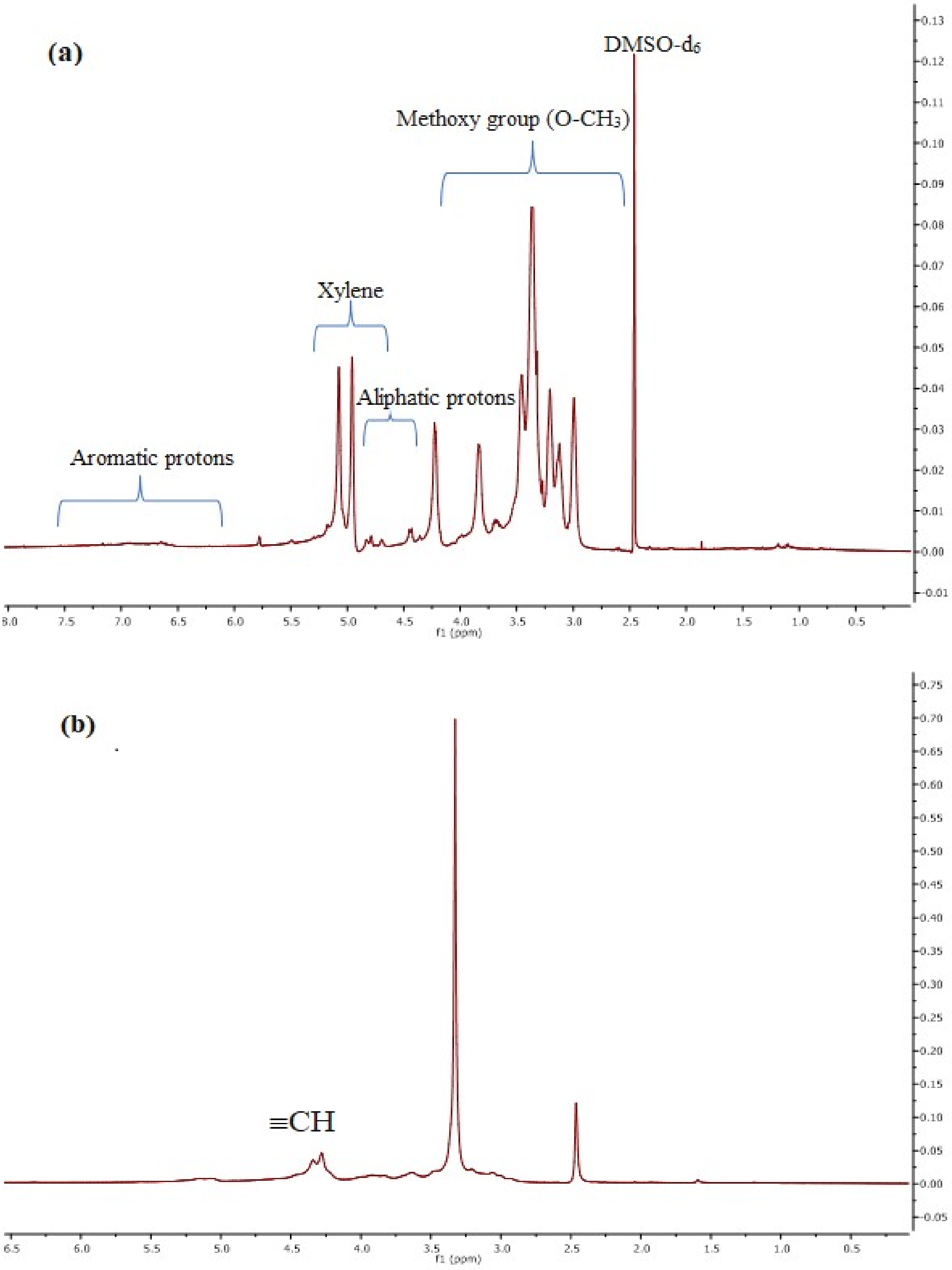

Fig. 6. ^{1}H NMR spectrum of lignin (a) and propargylated lignin (b).

5. Water contact angle measurements

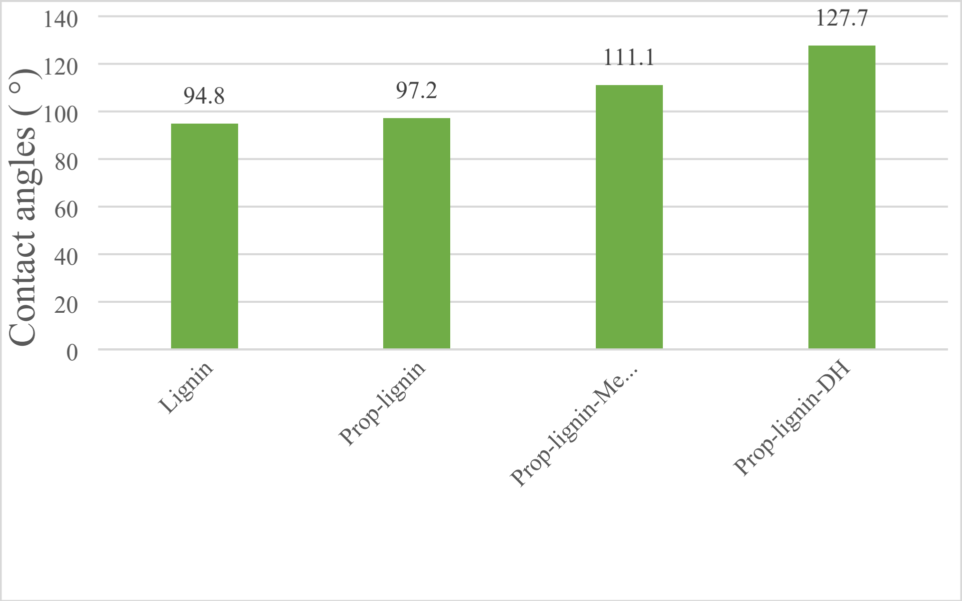

Fig. 7. Main contact angle data for the prepared samples.

6. Solubility measurements

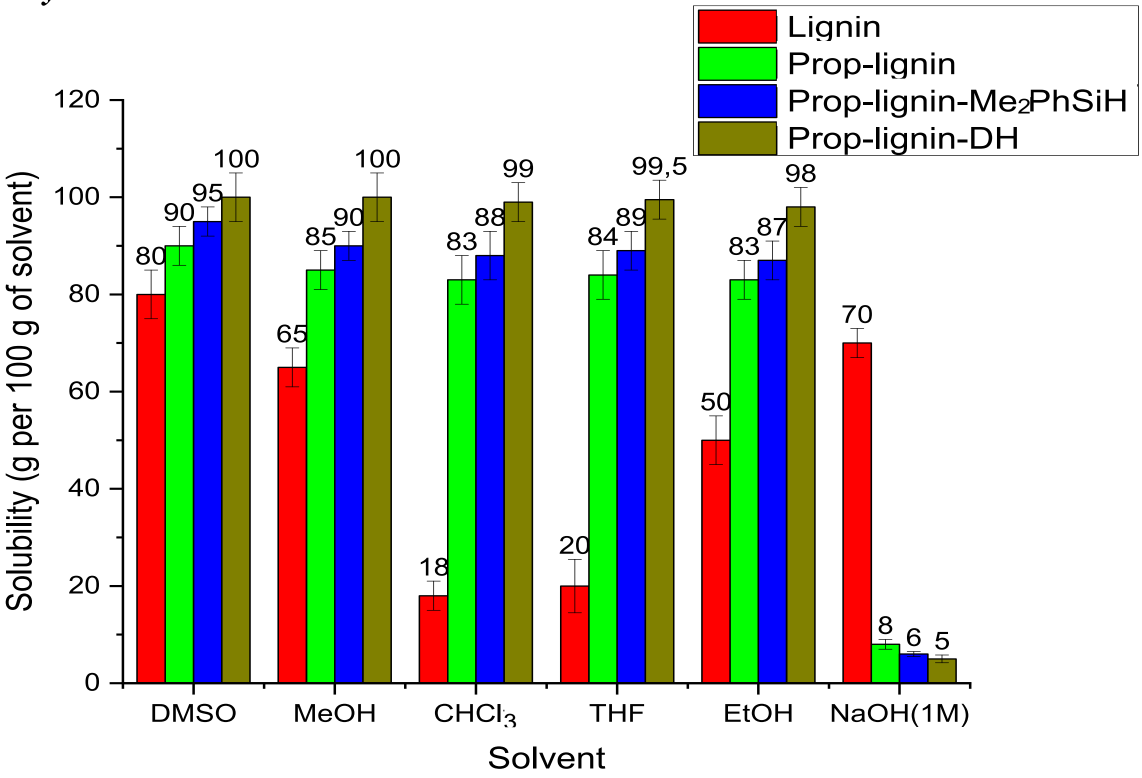

Fig. 8. Solubility diagram of lignin and modified lignin in various solvents at 25°C.

7. Formulated resins characterization

Table 3. Resin physicochemical characteristics at different loading rates.

Adhesives		pH	Solid content, (%)	Gel time at 120°C, *(s)*	Viscosity, *(cP)*
Control PF	0//100	11.20	43	966	417
lignin//PF	5//95	11.18	47	817	492
	7//93	11.17	51	774	524
	10//90	11.15	54	710	579
	15//85	11.14	59	677	752
Prop–lignin–DH//PF	5//95	11.21	49	829	490
	7//93	11.19	52	781	554
	10//90	11.17	54	695	639
	15//85	11.16	60	661	721

8. Resin bond strength and wood failure

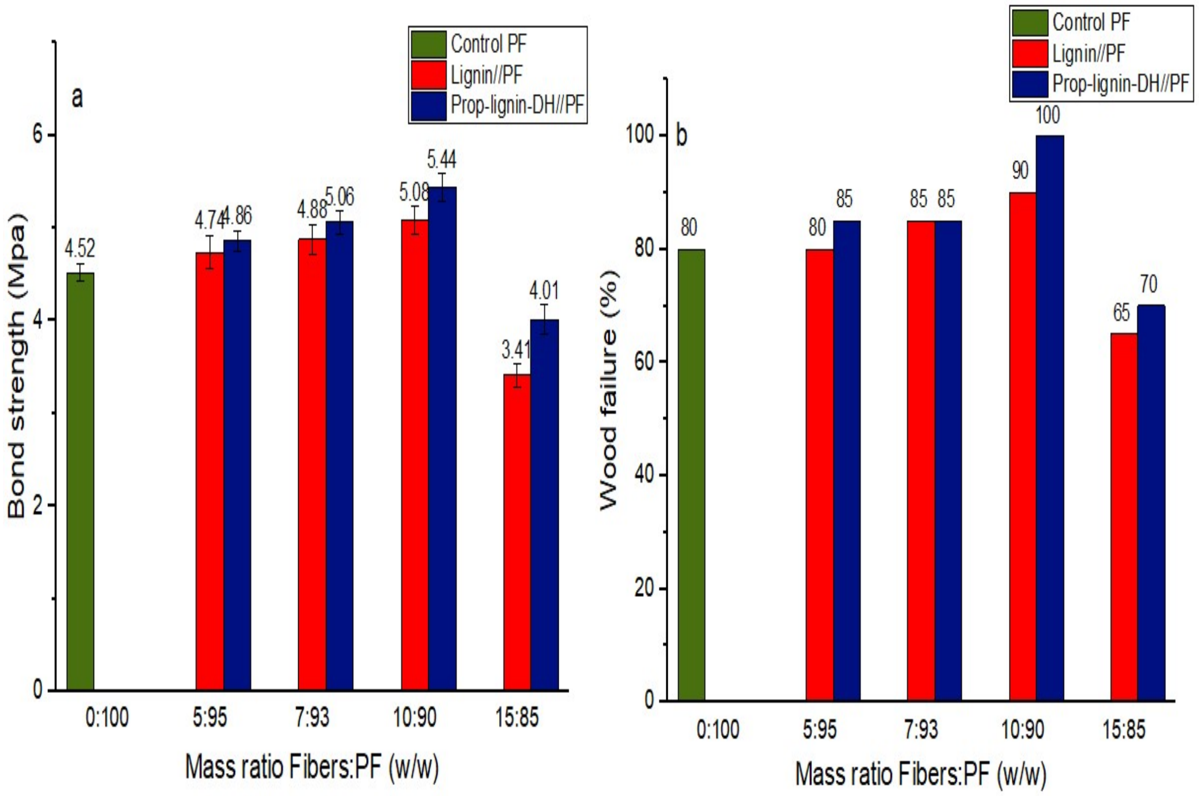

Fig. 9. Bond strength (a) and wood failure (b) of plywood with two layers, bonded using control PF resin, lignin-PF resin, and Prop-lignin-DH resin at different loading ratios.

9. Panel mechanical properties *and* Emission of formaldehyde

Table 4. SS, MOR, MOE and FE of plywood panels glued with control PF, optimal resins lignin-PF and Prop-lignin-DH-PF.

Plywood panels	Shear strength mean ± SD (N/mm^2)					MOR Mean ± SD (N/mm^2)	MOE Mean ± SD (N/mm^2)	Formaldehyde emission Mean ± SD ($mg/m^2/h$)
	Dry	Cold water at 20 °C for 24 h	Boiling Water after					
			3 h	6 h	9 h			
Control PF (0:100)	1.80±0.08	1.71±0.08	1.52±0.10	0.97±0.14	0.54±0.07	48±2.09	3258±75	2.51±0.26
Lignin :PF 10:90 (w/ w)	2.04±0.15	1.82±0.10	1.51±0.08	1.21±0.12	0.80±0.07	57±1.39	3605±83	2.31±0.12
Prop-lignin-DH :PF 10:90 (w/ w)	2.81±0.11	2.01±0.09	1.75±0.07	1.49±0.10	0.94±0.08	66±1.02	4148±81	2.01±0.13
GB/T 17657-2013	> 0.7	> 0.7						
EN 16352	> 1							
EN310						20.2	2280	
EN13986:200 E1								≤ 3.5
EN13986:2005 E2								≤ 8
